# Artemisinin derivatives induce oxidative stress leading to DNA damage and caspase-mediated apoptosis in *Theileria annulata*-transformed cells

**DOI:** 10.1186/s12964-023-01067-7

**Published:** 2023-04-17

**Authors:** Madhumanti Barman, Debabrata Dandasena, Akash Suresh, Vasundhra Bhandari, Sonam Kamble, Sakshi Singh, Madhusmita Subudhi, Paresh Sharma

**Affiliations:** 1 National Institute of Animal Biotechnology, Hyderabad, India; 2 Graduate Studies, Regional Centre for Biotechnology (RCB), Faridabad, India; 3 Present Address: National Institute of Pharmaceutical Education and Research (NIPER), Hyderabad, India

**Keywords:** Artemisinin, *Drug* repurposing, Oxidative DNA damage, DNA double-stranded breaks, Apoptosis, Apicomplexan

## Abstract

**Background:**

Bovine theileriosis caused by the eukaryotic parasite *Theileria annulata* is an economically important tick-borne disease. If it is not treated promptly, this lymphoproliferative disease has a significant fatality rate. Buparvaquone (BPQ) is the only chemotherapy-based treatment available right now. However, with the emergence of BPQ resistance on the rise and no backup therapy available, it is critical to identify imperative drugs and new targets against *Theileria* parasites.

**Methods:**

Artemisinin and its derivatives artesunate (ARS), artemether (ARM), or dihydroartemisinin (DHART) are the primary defence line against malaria parasites. This study has analysed artemisinin and its derivatives for their anti-*Theileria*l activity and mechanism of action.

**Results:**

ARS and DHART showed potent activity against the *Theileria-*infected cells. BPQ in combination with ARS or DHART showed a synergistic effect. The compounds act specifically on the parasitised cells and have minimal cytotoxicity against the uninfected host cells. Treatment with ARS or DHART induces ROS-mediated oxidative DNA damage leading to cell death. Further blocking intracellular ROS by its scavengers antagonised the anti-parasitic activity of the compounds. Increased ROS production induces oxidative stress and DNA damage causing *p53* activation followed by caspase-dependent apoptosis in the *Theileria-*infected cells.

**Conclusions:**

Our findings give unique insights into the previously unknown molecular pathways underpinning the anti-*Theileria*l action of artemisinin derivatives, which may aid in formulating new therapies against this deadly parasite.

**Video abstract**

**Supplementary Information:**

The online version contains supplementary material available at 10.1186/s12964-023-01067-7.

## Introduction

Apicomplexan parasites cause a significant economic loss in humans and animals worldwide. The tick-borne parasite *T. annulata* is one such significant intracellular pathogen that affects the productivity and health of the livestock [1]. The disease is life-threatening and can cause animal death if not treated promptly. The parasite infects the host macrophages and lymphocyte cells, resulting in severe lymphoproliferative disease [2]. The *Theileria-*transformed cells acquire cancer-like hallmarks such as uncontrolled cell proliferation, immortality, and metastasis [3, 4]. This disease has reported an economic loss of US$ 1295 million annually in India [5, 6]. BPQ is presently the first-line treatment for all *Theileria* parasites. It is the only drug used for treating bovine theileriosis (BT). However, reports of treatment failure have surfaced recently, pointing to the need for alternative treatments to curtail the disease [7–9]. Hence, there is an urgent need to identify new drugs and targets for treating BT [10, 11]. Natural compounds would be advantageous for new or repurposed therapy because of their minimal host toxicity, side effects, and cost.

Artemisinin (ART) is a naturally derived plant compound that is highly efficient in killing all species of *Plasmodium*. WHO recommends artemisinin and its derivatives (artesunate, artemether, or dihydroartemisinin) to treat malaria parasites in endemic countries [12]. ART and its derivatives have shown a marked decrease in malaria-associated mortalities [13]. Other than *Plasmodium* parasites, artemisinin and its derivatives have shown promising anti-parasitic activity against multiple other human and animal parasites, such as *Trypanosoma spp., Leishmania spp., Naegleria fowleri, Acanthamoeba castellanii, Giardia lamblia, Eimeria spp., Toxoplasma gondii, Cryptosporidium parvum, Babesia spp.,* and *Neospora caninum* [14–32]. In vitro and in vivo studies have shown that artemisinin and its derivatives are also efficient in treating many different types of cancers [33, 34].

Since *Theileria* belongs to apicomplexan parasites and is known to induce cancer-like features in the host cells, we thought of studying the anti-*Theileria*l activity of these compounds [3]. Therefore, our present study aims to repurpose the malarial drugs to evaluate their anti-parasitic activity against the schizont stage *T. annulata* parasite. We have also deciphered the action mechanisms of these drugs by checking ROS generation, oxidative DNA damage, DNA double-stranded breaks, apoptosis, mitochondrial potential, and p53 expression. This study will help in finding new treatment options against BT.

## Materials and methods

### *Theileria annulata* parasite culture and drug susceptibility assay*:*

*Theileria annulata-*infected cells were cultured in RPMI 1640 medium with 10% FBS, and the drug susceptibility testing were performed as previously reported [35]. Similarly, host cell cytotoxicity experiments were carried out utilising PBMCs isolated from seemingly healthy cattle that tested negative for *T. annulata* infection. Each experiment was performed five times. A survival test was conducted to assess the ability of *Theileria*-infected cells to withstand drug pressure. The cells were treated with ARS, DHART, and BPQ for 48 h. The cultures were washed with RPMI media and restored to normal conditions at the conclusion. The viability of the cells was checked every 72 h. The cultures were observed for 12–14 days for live cells. Artemisinin (ART), ARS, DHART, and ARM were purchased from Cayman chemicals. BPQ (B4725) was purchased from Sigma. All the compounds were prepared as 1 mg/mL stock solution in 100% DMSO.

### Checkerboard assay

The checkerboard assay was performed to evaluate the synergistic effect of the ARS and DHART drugs in combination with BPQ. The cells were incubated with increasing concentrations of drugs ARS (0–50 µg/mL) and BPQ (0–50 ng/mL) or DHART (0–50 µg/mL) and BPQ (0–50 ng/mL) to find the synergy between the compounds based on the Fractional Inhibitory Concentration (FIC) Index. For ARS and DHART, we made two-fold serial dilutions of ARS beginning at 50 µg/mL, 25, 12.5, 6.25, 3.125, 1.56–0.78 µg/mL and for BPQ twofold serial dilutions beginning at 50 ng/mL, 25, 12.5, 3.125, 1.56, 0.78, 0.39, and 0.195 ng/mL.

After 48 h, fluorescence data were analysed using Compusyn software [36, 37]. Results of the checkerboard assay were interpolated based on FIC values as follows: S, synergy (FIC ≤ 0.5); A, additive (FIC > 0.50 and < 1); I, indifferent (FIC > 1 and ≤ 4) [37].

### Proliferation assay

A time-course experiment was set up for 48 h to assess the time-dependent effect of drug treatment on the proliferation of the *Theileria-infected* cells. The proliferation experiment was performed in a 6-well plate with 1 × 10^6^ cells per well. The infected cells were treated with the IC_50_ value of the individual compounds, and the concentration was decided based on the FIC values for the wells receiving drug combinations. After drug treatment, cells were collected and counted every 12 h till 48 h by trypan blue assay as per standard protocol. The untreated cells were used as a control. The proliferation experiment with the compounds was performed five times in triplicates. The graph represents the average data from all five experiments.

### Comet assay for DNA damage

Comet assay was used to measure the DNA damage in the *T. annulata* infected cells. The cells were incubated with 1× IC_50_ of the compounds for DNA damage analysis. The treated and untreated cells were diluted in DPBS and mixed with 1% low melting agarose. This cell suspension was then loaded on 1% high melting agarose-coated slides and incubated for a few minutes at 4 °C as per standard protocol [38]. To lyse the cells, the slides were then dipped in an alkaline lysis solution for 16 h at 4 °C followed by electrophoresis at 40 V. Further slides were fixed with 70% ethanol and stained with SYBR green dye. After staining, images were taken in an Apotome microscope, and the Olive tail moment (OTM) was calculated by ImageJ software. OTM was calculated in the same manner as previously mentioned [38].

### Measurement of intracellular ROS and oxidative damage

The H2DCFDA dye (Invitrogen) was used for measuring intracellular ROS in the *Theileria-*infected cells. After treatment, the cells were collected at different time points to measure dose-dependent intracellular ROS production. Data acquisition and analysis were made using a BD flow cytometer and Flowjo software (Tree Star Inc., USA). Oxidative DNA damage was measured using the 8-OHdG (sc-393871) antibody (Santa Cruz Biotech) by doing an immunofluorescence assay (IFA) according to the previously reported method [39]. Mean fluorescence intensity was plotted for each cell, and one-way ANOVA was performed for multiple comparisons between the treated sample and the control.

### Cell death and apoptosis assay

Briefly, *Theileria* infected cells were treated with test drugs (IC_50_), and cells were collected at different time intervals (0, 6, 12, 24, 36, and 48 h) till 48 h. The untreated cells were taken as control. Annexin V and propidium iodide (PI) based staining was done to measure the apoptosis and necrosis in the cells [35]. All the experiments were done in duplicate.

### Immunofluorescence assay and Western blot analysis

IFA experiments were done to evaluate the oxidative damage (8-OHdG), phosphorylation levels of the H2AX (Ser 139) and the localization of p53 in the *Theileria-*infected cells. Primary antibodies were: 8-OHdG (1:200), Phospho-Histone H2A.X (Ser139) antibody (1:300), p53 antibody (1:200) (Thermofisher), and rabbit anti-TaSP peptide antibody (1:200). The IFA experiment and analysis were performed as previously reported [35]. Briefly, 5 × 10^5^ *T. annulata* infected cells were cultured in the media containing IC_50_ concentration of drugs. Cells without drugs in the medium served as a control in the experiment. The negative control was BPQ-treated cells. After drug treatment, cells were pelleted and washed three times with 1X PBS. The cells were then fixed with 4% paraformaldehyde (37 °C,10 min), washed in PBS, and permeabilized with 0.1% Triton X-100. Permeabilized cells were incubated for 1 h at room temperature in a blocking buffer (2% BSA in 1X PBS). The cells were then incubated with primary antibodies overnight at 4 °C. To remove the unbound primary antibody, cells were thoroughly washed in PBS containing 0.05% Tween20. After washing, cells were incubated for 1 h at room temperature with a secondary antibody. The cells were then washed, and the DNA was labelled with DAPI. After mounting the samples with mounting media, images were captured using a Zeiss fluorescent microscope with a 100× objective. The ZEN 3.3 (blue edition) programme was used for image processing and quantification.

The time-dependent activation of p53 in *Theileria*-infected cells was evaluated by Western blotting. For western blot analysis, cell lysates from time course studies (0, 6, 12, 24, and 48 h) were collected and suspended in the radioimmunoprecipitation assay (RIPA) buffer. SDS-PAGE and western blot analysis was performed on the collected samples using a Phospho-p53 (Ser15) primary antibody, and an Anti-IgG HRP conjugated rabbit secondary antibody (1:2000). As a control, host HRP conjugated β-actin (1:5000) was utilised to ensure that the protein samples were loaded equally. Western blot images were developed using the Bio-Rad ChemidocTM Imaging system with a chemiluminescent HRP substrate.

### Cell cycle and mitochondrial potential analysis

*Theileria annulata* infected cells were treated individually and in combination with the IC_50_ values of the compounds, untreated cells served as control. Cells were collected 12 and 24 h after treatment, followed by washing with DPBS. The washed cells were incubated with 500 µL of cell staining buffer (50 µg/mL Propidium iodide PI, 0.1% Triton-X, and 200 µg/mL RNase in DPBS) at 37 °C for 20 min. The cell cycle distribution and analysis were done by using BD flow cytometry and FlowJo X.

*Theileria annulata*-infected cells were incubated with a JC-1 probe to measure mitochondrial membrane potential before and after treatment with compounds as described before [35]. Cells treated with staurosporine served as a control. The BD LSR Fortessa was used for collecting data, and Flow Jo was used for analysis (Tree Star Inc., Ashland, OR). Depolarization of mitochondria was measured by dividing the intensity of fluorescence emission in red by fluorescence emission in green. All the experiments were done in duplicate. Ordinary one-way ANOVA was performed to check the statistical significance, and Dunnett's multiple comparisons test was used for the analysis. The mean of each treatment was compared with the control, and the graph represents the standard error mean.

### Scanning electron microscopy (SEM)

SEM was performed on *Theileria*-infected cells with and without treatment with drugs. The fixation and preparation of cells for SEM was done as described earlier [40]. Briefly, the dried treated and untreated cells were coated with gold using the sputter coater and were analysed with Zeiss ULTRA 55 SEM.

## Results

### Artemisinin derivatives showed potent anti-*Theileria*l activity

**Fig. 1:**
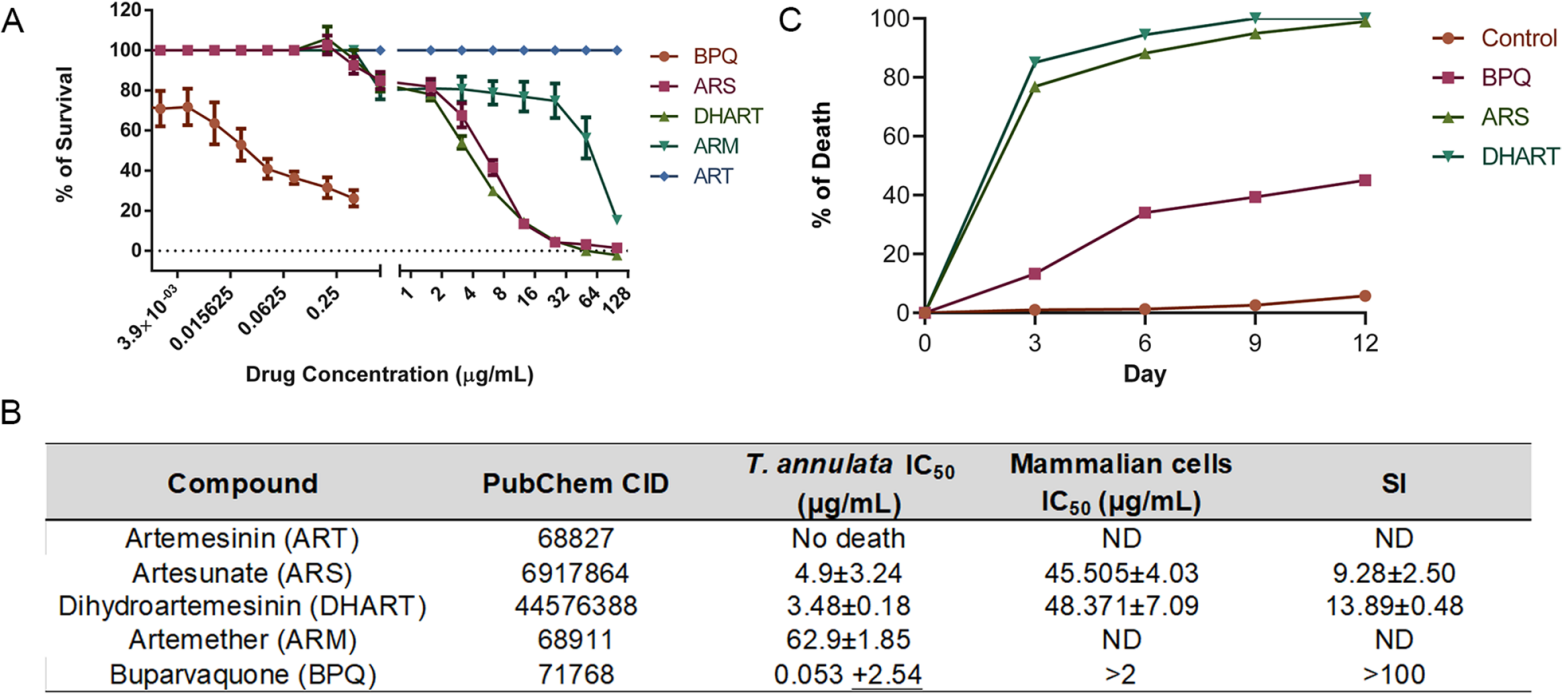
Artemisinin derivatives showed potent anti-*Theileria*l activity. **A** The graph shows the percentage of growth inhibition of *T. annulata* infected cells treated with different concentrations of Artemisinin and its derivatives (artesunate, artemether, or dihydroartemisinin) after 48 h of treatment. BPQ, a potent anti-*Theileria*l drug, was used as a positive control. **B** In vitro efficacies of Artemisinin and its derivatives against *T. annulata-*infected cells. PBMCs isolated from healthy cattle were used for calculating the host cytotoxicity. **C** The graph shows the effect of ARS, DHART, and BPQ after 12 days of culture

Artemisinin and its derivatives like ARS, DHART, and ARM were tested for their anti-parasitic activity against the *T. annulata* parasites. In vitro challenge studies showed the potent activity of the artemisinin derivatives against the *T. annulata* parasites except for artemisinin. DHART, with the lowest IC_50_ value, 3.48 µg/mL, showed the most potent activity, followed by ARS (4.9 µg/mL) and artemether (62.9 µg/mL). BPQ (IC_50_ = 50 ng/mL) was used as a positive control for all the experiments (Fig. 1A, B). The cell cytotoxicity studies using ARS and DHART with PBMCs from healthy cattle showed no host cytotoxicity against the compounds at their IC_50_ concentrations (Fig. 1B).

The survival assay to check the drug tolerance capacity of the *Theileria-infected* cells showed complete growth suppression and the irreversible effect of ARS and DHART. We noticed complete clearance of the cells in the DHART or ARS-treated cells compared to the BPQ (Fig. 1C).

### Artemisinin derivatives show early parasite clearance and synergism in combination with BPQ

**Fig. 2:**
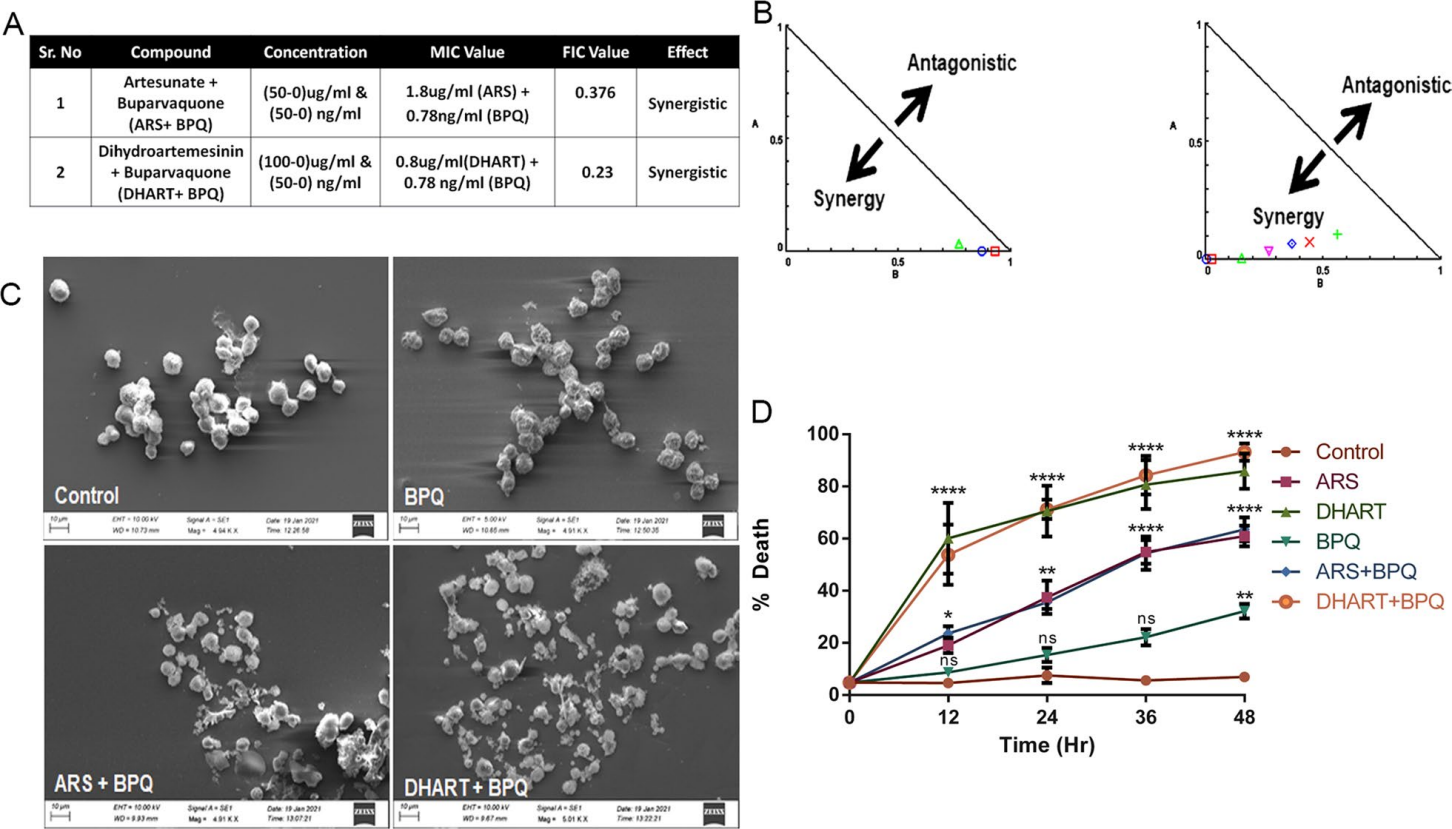
Artemisinin derivatives show early parasite clearance and synergism in combination with BPQ. **A** Table shows result of checker board assay using different combinations of artesunate, dihydroartemisinin, and BPQ against *T. annulata* infected cells after 48 h of treatment. **B** Graph shows the synergistic activity of the combination therapy. **C** Scanning electron microscopy showing the effect of combination therapy on *T. annulata* infected cells. **D** The graph shows the growth kinetics of treated and nontreated *T. annulata* infected cells using the trypan blue dye exclusion method. The data represent a total of 5 biological replicates for each time point. two-way ANOVA was performed to check the statistical significance, and Dunnett's multiple comparisons test was used for the analysis. The mean of each treatment was compared with the respective control time point, and the graph represents the standard error mean for each condition. In the graph, *represents the significance of (*P* ≤ 0.05), while **represents the significance of (*P* ≤ 0.01), ***represents the significance of (*P* ≤ 0.001), and ****represents the significance of (*P* < 0.0001)

ARS and DHART are effective against *T. annulata* infected cells; however, their IC_50_ values indicate that they are not as potent as BPQ. Therefore, we performed pharmacological combination studies to look for any interactions between BPQ and ARS or DHART. When combined with BPQ, ARS and DHART had a synergistic effect on *T. annulata* infected cells, as determined by mean FIC (synergy (FIC ≤ 0.5) values of 0.376 and 0.23, respectively (Fig. 2A, B). The parasite-killing effectiveness of BPQ increased by 64-fold in combination with ARS or DHART. Due to the synergistic nature of the effect, the total dosage of BPQ required for treatment is considerably less than when used alone. (Fig. 2B). The drug combination experiments on healthy PBMC cells demonstrated that the drugs (FIC concentration) did not affect control cells (Fig. 2A).

Significant morphological alterations were seen by SEM in cells treated with ARS or DHART in combination with BPQ after 48 h of treatment. The cell membranes of the control group were found to be intact. However, cells treated with the combination exhibited surface protrusions and cell membrane damage (Fig. 2C).

Then, we examined the impact of ARS or DHART therapy on the proliferation of *T. annulata*-infected cells as a function of time. The 48-h time course experiment demonstrated that the anti-proliferative impact of ARS or DHART, alone or in combination with BPQ, started as early as 12 h post-treatment (Fig. 2D). Furthermore, the anti-proliferative impact was much more significant and faster in ARS or DHART treatment than in BPQ alone. The time-course experiment indicated that ARS or DHART-based combination treatments are more potent and effective in killing *T. annulata*-infected cells than BPQ alone.

### Artemisinin derivatives cause DNA damage and DNA double-strand breaks (DSBs) in the *T. annulata* infected cell

**Fig. 3:**
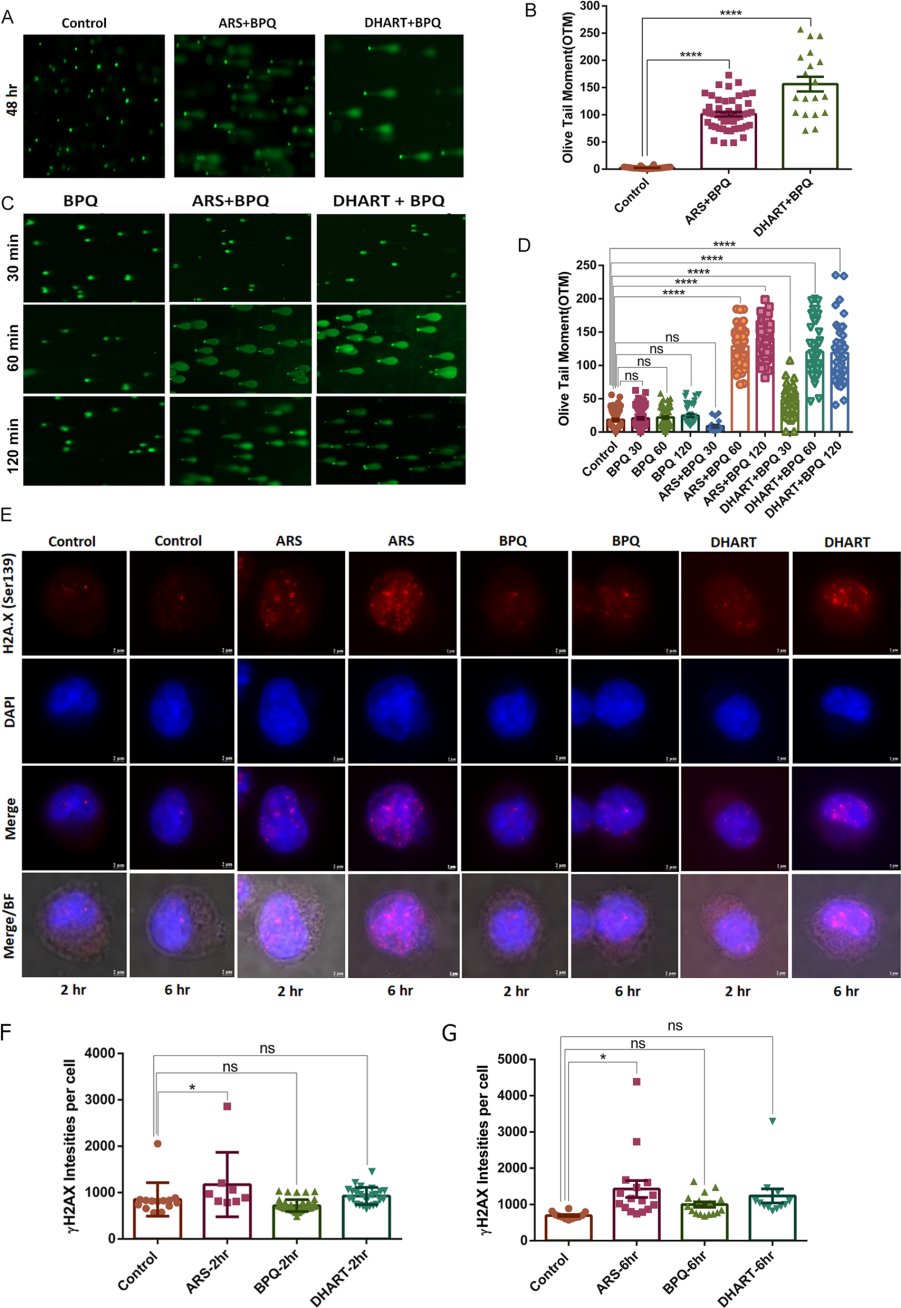
Artemisinin derivatives cause DNA damage and DNA double-strand breaks (DSBs) in the infected cell. **A** Comet assay visualisation of DNA damage in *T. annulata*-infected cells after 48 h of treatment with ARS + BPQ and DHART + BPQ, as compared to untreated parasites. **B** Comet assay evaluation of the olive tail moment (OTM) after 48 h with ARS + BPQ and DHART + BPQ, compared to untreated cells (Control). The error bars reflect mean plus/minus standard deviations. **C** Comet assay visualisation of DNA damage in *T. annulata* infected cells after 30, 60, and 120 min of treatment with ARS + BPQ and DHART + BPQ, relative to untreated parasites. **D** Comet assay assessment of the olive tail moment (OTM) after 30, 60, and 120 min with ARS + BPQ and DHART + BPQ against untreated (Control) cells. The error bars reflect mean standard deviations. **E** Immunofluorescence analysis of *T. annulata* infected cells after 2 h and 6 h of treatment with ARS, DHART, and BPQ using an anti-γ-H2A.X antibody (phosphorylated). DAPI was used for nuclear staining (blue). Ordinary 1way ANOVA was performed to check the statistical significance, and Dunnett's multiple comparisons test was used for the analysis. The mean of each treatment was compared with the control, and the graph represents the standard error mean. In the graph, ****represents the significance of (*P* < 0.0001), ns represents nonsignificant results with *P* value > 0.05 while *represents the significance of (*P* ≤ 0.05)

Since ARS and DHART showed significant activity against *T. annulata-*infected cells, we attempted to understand their mechanisms of action. Researchers have previously linked the antimalarial effect of ARS to DNA damage in *Plasmodium* parasites [41]. Next, we examined DNA damage in *T. annulata*-infected cells treated with ARS or DHART in conjunction with BPQ using the alkaline comet assay. DNA damage in *Theileria*-infected cells treated with ARS or DHART was time-dependent. In agreement with the OTM findings, the comet assay demonstrated substantial DNA damage in treated cells after 48 h of treatment with both drugs (IC_50_) compared to untreated cells (Fig. 3A, B). Significant variations were seen in the proportion of tail movement values (OTM) between the BPQ-only and combination treatment groups. DNA Damage was apparent in treated cells just after 60 min of treatment (Fig. 3C, D). After 48 h of treatment, we withdrew the pharmacological drug pressure and attempted to grow the parasites; however, there was no recovery or growth in ARS or DHART-treated cells, suggesting permanent DNA damage [42]. However, after BPQ removal, parasites could recuperate and multiply in the medium.

Therefore, we explored whether exposure to ARS or DHART results in DSBs in *Theileria*-infected cells. DSBs were examined based on the phosphorylation level of the histone γH2AX protein, a typical indicator of DNA damage. The phosphorylation of the γH2AX protein increased after 2 and 6 h of ARS treatment, as shown by IFA analysis compared to uninfected and DHART or BPQ-treated cells (Fig. 3E–G).

### Artemisinin derivatives induce ROS-dependent DNA damage and oxidative stress in *Theileria-infected* cells

**Fig. 4:**
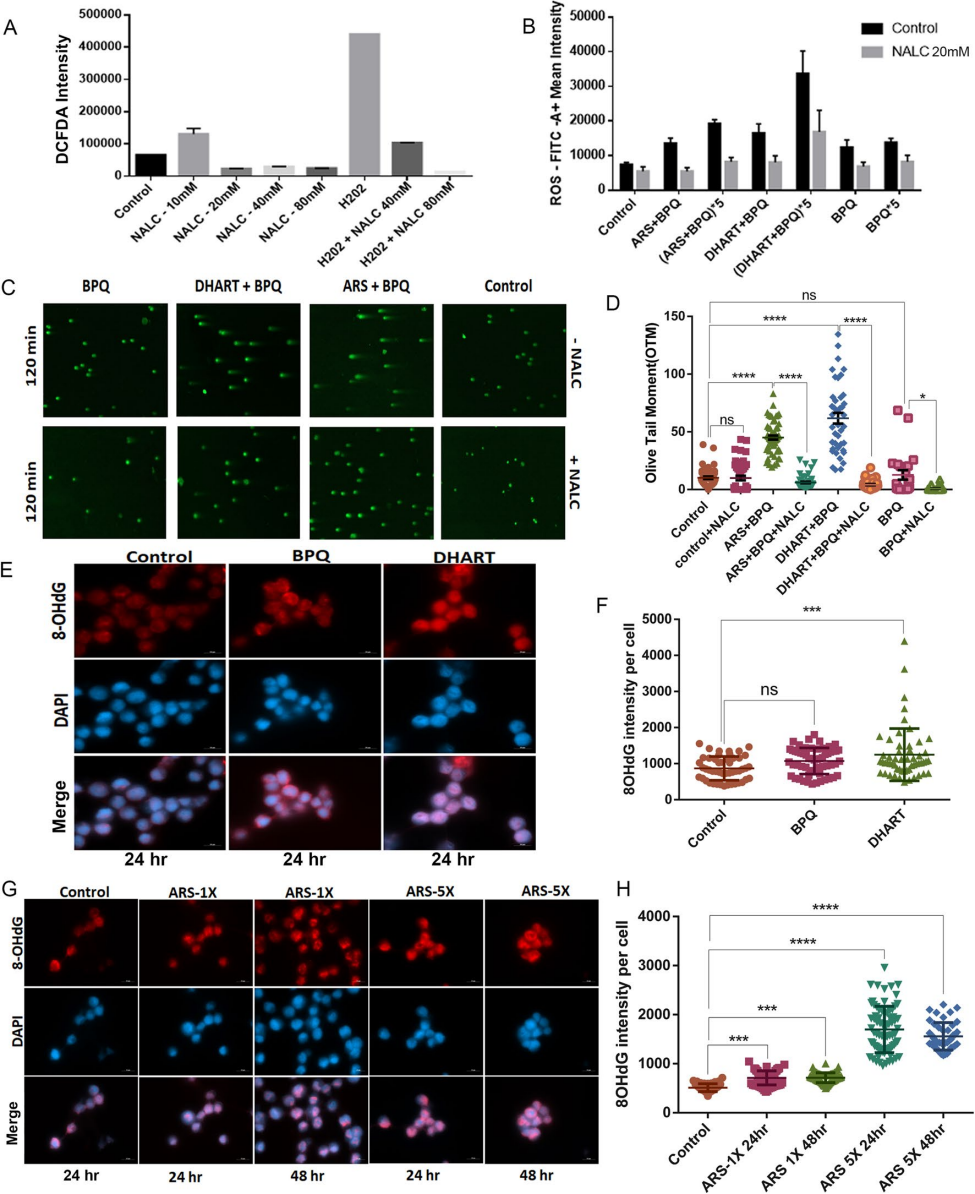
Artemisinin derivatives induce ROS-dependent DNA damage and oxidative stress in *Theileria-infected* cells: **A** Graph shows the relative fluorescence intensity of intracellular ROS produced in *T. annulata-infected* cells after treatment with H_2_0_2_ and different concentrations of NALC compared to untreated cells (Control). The error bars reflect mean plus/minus standard deviations. **B** The graph depicts the relative fluorescence intensity of intracellular ROS generated in *T. annulata* infected cells following treatment with various concentrations of ARS + BPQ and DHART + BPQ compared to untreated cells (Control) with or without NALC. The error bars reflect mean plus/minus standard deviations. **C** Comet assay visualisation of DNA damage in *T. annulata*-infected cells after 120 min of treatment with ARS + BPQ and DHART + BPQ, as compared to untreated parasites with or without NALC. **D** Comet assay evaluation of the olive tail moment (OTM) after 120 min of treatment with ARS + BPQ and DHART + BPQ, compared to untreated cells (Control) with or without NALC. Ordinary 1way ANOVA was performed to check the statistical significance and Tukey’s multiple comparisons test was used for the analysis. The mean of each group was compared with the control as well as with each other and the graph represent standard error mean. In the graph ****represents significance of (*P* < 0.0001) while ns represents nonsignificant results with *P* value > 0.05 while *represents significance of (*P* ≤ 0.05). The error bars reflect mean plus/minus standard deviations. **E** Immunofluorescence staining of 8-OHdG after 24 h of treatment with DHART; untreated and BPQ treated cells were used as a positive control. DAPI was used for nuclear staining. **F** The graph depicts the levels of 8-OHdG in *T. annulata* infected cells after 24 h of DHART treatment as measured by fluorescence intensity using the appropriate ZEN 3.3 program. **G** Immunofluorescence staining of 8-OHdG after 24 h and 48 h treatment with ARS at different concentrations (1× and 5×); untreated and BPQ treated cells were used as a positive control. DAPI was used for nuclear staining. **H** The graph depicts the levels of 8-OHdG in *T. annulata* infected cells after 24 h and 48 h treatment with ARS as measured by fluorescence intensity using the appropriate ZEN 3.3 program. A minimum of forty cells were counted for each experiment and measurement point. The error bars reflect mean plus/minus standard deviations. Ordinary 1way ANOVA was performed to check the statistical significance, and Dunnett's multiple comparisons test was used for the analysis in **F**, **H**. The mean of each group was compared with the control and each other, and the graph represents the standard error mean. In the **F** graph, ***represents the significance of (*P* = 0.0009) while ns represents nonsignificant results with a *P* value > 0.05. In **H**, ***represents the significance of (*P* ≤ 0.001), and ****represents the significance of (*P* < 0.0001)

Infected cells with elevated ROS levels have been shown to generate DNA double-strand breaks [42, 43]. We next wanted to investigate whether ARS or DHART treatment increases ROS production and oxidative stress and if so, if it is responsible for the death observed in *Theileria*-infected cells. NALC (N-acetyl-L-cysteine), a well-known antioxidant and negative regulator of ROS production, was used as a negative control. Cells treated with H_2_O_2_ were used as a positive control (Fig. 4A). The flow cytometry experiment revealed a substantial increase in intracellular ROS generation in samples treated with ARS or DHART alone or in combination with BPQ (Fig. 4B). The study revealed a concentration-dependent decrease in ROS production in cells treated with NALC, ARS, and DHART (Fig. 4B). Blocking intracellular ROS by its scavengers antagonises the anti-parasitic activity of the ARS and DHART. To investigate the effect of ROS production on DNA damage in *Theileria*-infected cells, a comet assay was conducted by incubating treated samples with and without NALC (20 mM) for 120 min. According to OTM values, increased ROS production in treated cells leads to DNA damage. However, no DNA damage was seen when the cells were co-incubated with NALC or BPQ alone, indicating that ROS-dependent DNA damage occurred in parasite-infected cells treated with ARS or DHART (Fig. 4C, D).

We next assessed the amount of 8-OHdG, the most common DNA oxidation product, since increased ROS may cause nuclear DNA damage [39]. To verify this theory, we evaluated the amounts of 8-OHdG in *Theileria*-infected cells by IFA analysis. As shown in (Fig. 4E, G) (for IFA) and (Fig. 4F, H) (for quantification), DHART and ARS induced a rise in 8-OHdG levels in the treated cells. In the first 24 h after the addition of DHART to the medium, the production of 8-OHdG rose dramatically (Fig. 4F). This increase, however, was not significant in ARS-treated cells compared to DHART. Using IC_50_ and 5X IC_50_ doses of ARS, we extended the experiment to 48 h. The levels of 8-OHdG started to increase substantially with time and the concentration of ARS (Fig. 4G, H).

Our results demonstrated that DHART and ARS therapy generated a persistent ROS level that accumulated throughout the treatment, leading to oxidative DNA damage. Moreover, in the presence of the ROS scavenger NALC, the impact of DHART and ARS in *Theileria*-infected cells was significantly reduced, indicating that the effect is a consequence of drug-induced oxidative DNA damage.

### Artemisinin derivatives induce cell cycle arrest and apoptosis

**Fig. 5:**
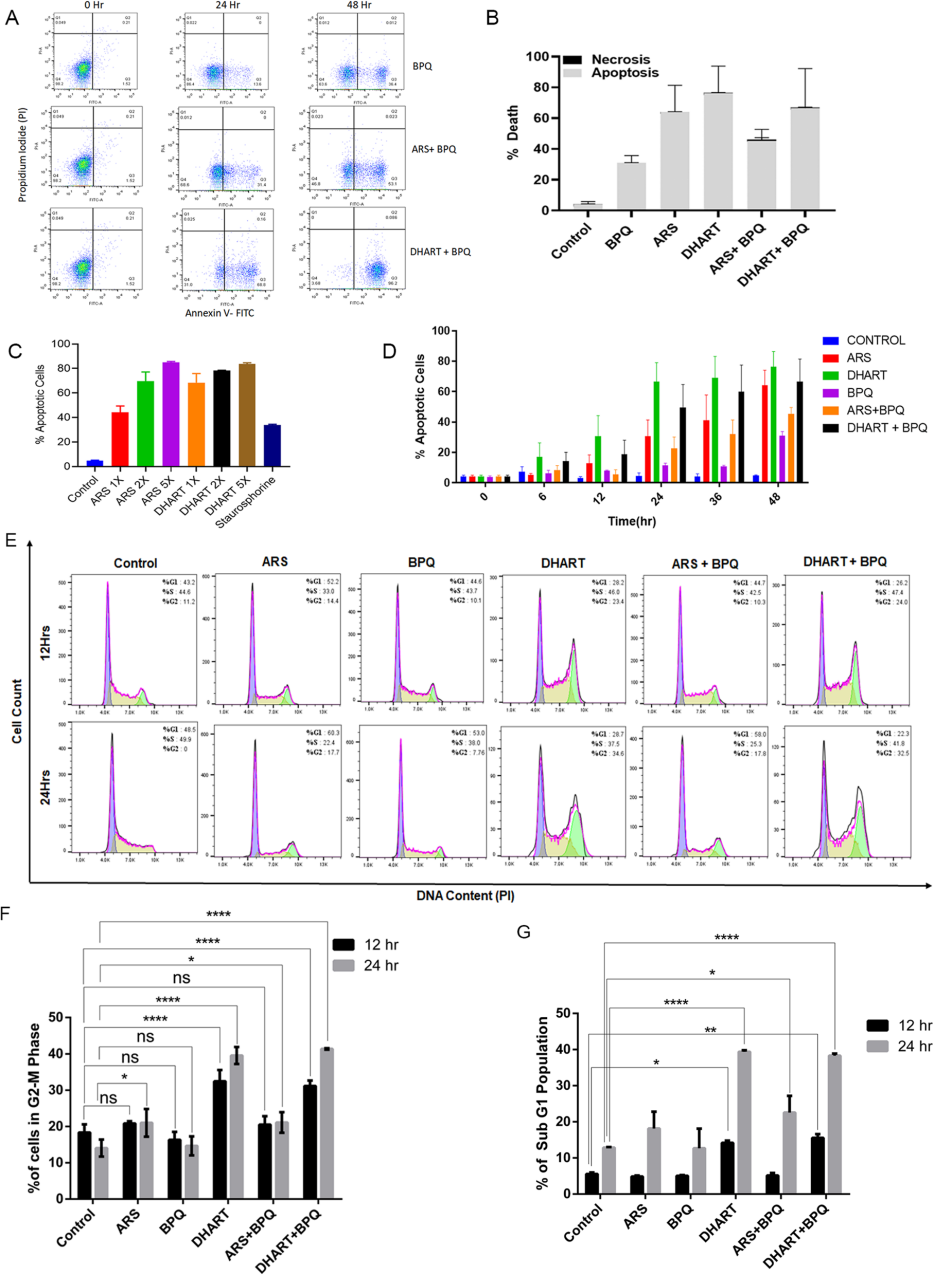
Artemisinin derivatives induce cell cycle arrest and apoptosis-mediated cell death: **A** representative flow cytometry analysis of *Theileria* infected cells with or without treatment. Induced cell death was determined 24 h and 48 h after treatment with ARS + BPQ and DHART + BPQ compared to BPQ treated cells (Control) by Annexin V/PI based flow cytometry analysis. **B** The graph shows the percentage of apoptosis and necrosis in the *T. annulata* infected cells after 48 h of treatment with different compounds. **C** The graph shows the percentage of apoptotic cells after treatment with various concentrations of compounds based on the FACS (Annexin V/PI) analysis. Staurosporine (known apoptosis inducer) treated sample was used as a positive control. **D** The graph shows the percentage of apoptotic cells after treatment with different compounds based on the FACS (Annexin V/PI) analysis at different time intervals. BPQ-treated sample was used as a positive control. **E** Representative flow cytometry analysis to determine the cell cycle distribution of *Theileria*infected cells after 12 and 24 h of treatment with compounds based on the PI staining. **F** The graph shows the percentage of G2-M population cells after 12 and 24 h of treatment with compounds based on the FACS (PI) analysis. The error bars reflect mean plus/minus standard deviations. **G** The graph shows the percentage of sub-G1 population cells after 12 and 24 h treatment with compounds based on the FACS (PI) analysis. BPQ-treated sample was used as a positive control. The error bars reflect mean and standard deviations. The comparisons were performed between the treated samples with the control sample for the same time points (12 h vs 12 h and 24 h vs 24 h). For multiple comparisons, 2-way ANOVA was performed. For 5F—single *(*P* value = 0.0301), ****(*P* value < 0.0001) [Sidak's multiple comparisons test]. while ns represents nonsignificant results with a *P* value > 0.05. For 5G—single *(*P* ≤ 0.05), **(*P* ≤ 0.01), ****(*P* < 0.0001) [Dunnett's multiple comparisons test]. while ns represents nonsignificant results with a *P* value > 0.05

DNA damage caused by DSBs may trigger cell death and apoptosis. Using FACS, the apoptotic markers propidium iodide (PI) and annexin V were assessed in *Theileira*-infected cells treated with ARS or DHART with BPQ. The time kinetics experiment demonstrated a significant increase in annexin V-positive cells after treatment with DHART or ARS (Fig. 5A, B). Cells treated with ARS and DHART exhibited a concentration and time-dependent increase in early apoptosis (Fig. 5C, D).The experiment revealed that treatment with DHART or ARS promotes apoptosis within 12 to 24 h in *Theileria*-infected cells. The annexin V-positive population increased from 2% under standard settings to 68.8% and 31% after 24 h treatment with DHART and ARS, respectively (Fig. 5A). In contrast, apoptosis (36%) was not detected in BPQ-treated cells based on the annexin V staining until 48 h post-treatment. According to the PI staining, there was no necrosis following drug treatment (Fig. 5A, B).

To further study the inhibitory effects of ARS, DHART, and BPQ on the proliferation of *Theileria*-infected cells, the cell cycle distribution was studied 12 and 24 h after treatment. The impact of drug treatment on cell cycle distribution was investigated using PI labelling (flow cytometer), and the findings are presented in (Fig. 5E–G). *Theileria*-infected cells exposed to DHART (IC_50_) for 12 h exhibited a substantial increase in G2/M phase cells, suggesting cell cycle arrest and early death. The G2/M phase cells were unaffected by ARS and BPQ treatments. Then, we evaluated cell death by assessing the sub-G1 population, which reveals that DHART, in conjunction with BPQ, triggers cell death as early as 12 h and rises by 24 h. However, no such increase in the subG1 population was detected with BPQ or ARS alone; although, the combination of ARS + BPQ at 24 h generated a considerable increase in subG1 population-driven cell death. The cell cycle data further validates our annexin V findings, revealing early apoptotic events with DHART, whereas ARS and BPQ fail to induce apoptosis until 24 h after treatment. These findings indicate that DHART may effectively stop the G2/M phase of the cell cycle and initiate early death in infected cells within 12 h of treatment.

### Artemisinin derivatives activate caspase-dependent mitochondrial apoptotic pathways

**Fig. 6:**
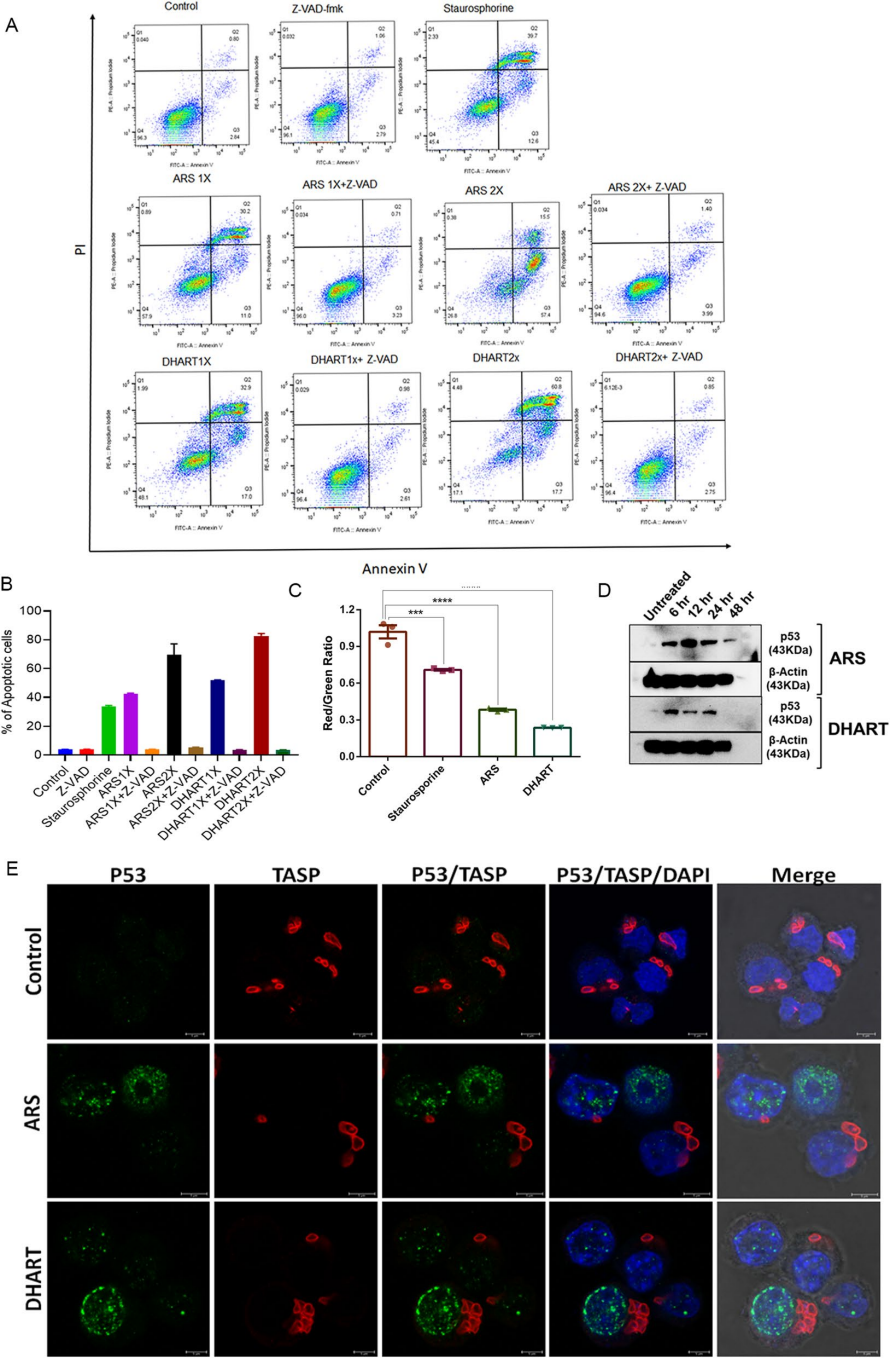
Artemisinin derivatives activate caspase-dependent mitochondrial apoptotic pathways **A** Representative flow cytometry analysis of *Theileria *infected cells with or without treatment. Induced cell death was determined 48 h after treatment with ARS or DHART in combination with or without Z-VAD-fmk by Annexin V/PI-based flow cytometry analysis. **B** The graph shows the percentage of apoptotic cells after treatment with various concentrations of ARS or DHART based on the FACS (Annexin V/PI) analysis. Staurosporine treated sample was used as a positive control. The error bars reflect mean plus/minus standard deviations. **C** The graph shows the assessment of mitochondrial potential using JC-1 dye based on the red (~ 590 nm)/green (~ 529 nm) fluorescence intensity ratio of *Theileria-infected* cells with or without treatment. Staurosporine treated sample was used as a positive control. The error bars reflect mean plus/minus standard deviations. In the graph, ****represents the significance of (*P* < 0.0001), while ***represents the significance of (*P* < 0.001). **D** Western blot analysis of the ART and DHART treated cells using p53 and ß-actin antibodies. **E** Immunofluorescence analysis of *T. annulata* infected cells after 24 h treatment with ARS and DHART using anti-p53 and TaSP antibody. DAPI was used for nuclear staining

To explore the involvement of caspases in apoptosis, infected cells were pre-treated with the cell-permeable pan-caspase inhibitor of apoptosis, z-VAD-FMK, before being challenged with DHART or ARS. Additionally, we analysed the treated cells for other characteristics linked with the apoptosis-like activation/nuclear localisation of the p53 and reduction in mitochondrial potential. Caspase activity and mitochondrial potential (JC-1 dye) were evaluated using flow cytometry. Caspase activity was measured 24 h after infected cells were treated with DHART or ARS at IC_50_ values (1× and 2×) with or without z-VAD-FMK. Intriguingly, treatment with z-VAD-FMK could reverse the anti-*Theileria*l activity of both DHART and ARS. After exposure to DHART or ARS with z-VAD-FMK, there was a considerable reduction in the number of annexin V-positive cells. These results demonstrate that the induction of apoptosis in treated cells is caspase-dependent. Cells treated with z-VAD-FMK and staurosporine served as the experimental control (Fig. 6A, B).

In line with this observation, we next measured JC-1 absorption 24 h after treatment to evaluate the mitochondrial membrane potential of the infected cells. Using the red/green fluorescence intensity ratio of the JC-1 dye, the mitochondrial potential of the control cells was determined to be ~ 1.0 (Fig. 6C). After 24 h, cells treated with DHART or ARS exhibited a considerable drop in red fluorescence, resulting in a ratio of fluorescence intensity of 0.5, indicating loss of mitochondrial membrane potential (Δψm). Based on the JC-1 ratio, our findings indicate that parasite-infected cells treated with artemisinin derivatives undergo mitochondrial depolarization. Next, we investigated whether caspase-dependent apoptosis is connected to p53, which is important in regulating the DNA damage response, cell cycle arrest, and apoptosis in response to diverse cell stimuli [44]. Previously, employing BPQ, it was shown that apoptosis in *Theileria*-infected cells results in p53 activation and nuclear localization [4, 45–48].

We used western blot and IFA to determine p53 activation and localization. *T. annulata* infected cells were treated with ARS and DHART and probed with Phospho-p53 (Ser15) antibody for western blot analysis to determine p53 gene activation. p53 activation was found as early as 6 h in ARS or DHART-treated samples but not in untreated cells (Fig. 6D). However, the expression of p53 subsided by 48 h of treatment due to increased cell death. Finally, we used an immunofluorescence experiment to look at p53 localization. IFA with p53 and TaSP antibodies revealed that p53 was exclusively localized within the host nucleus, implying that it was activated and may have contributed to the apoptosis of parasite-infected cells (Fig. 6E). The exclusive localization of p53 inside the host cell's nucleus suggests that its transcriptional activity promotes apoptosis and cell death.

## Discussion

The treatment of BT heavily depends on the drug BPQ. Despite being the only available drug, it has issues such as drug resistance, side effects, and residual parasites in treated animals that act as lifelong disease carriers. Finding new drug targets with potent anti-parasitic activity is essential for controlling the *Theileria* parasites. The discovery of artemisinin and its derivatives is considered one of the path-breaking interventions in controlling the spread of malaria parasites. Artemisinin-based combination therapy is the standard treatment against uncomplicated and multi-drug-resistant malaria parasites in endemic countries [10]. Artemisinin and its derivatives are promising anti-cancer drugs due to their potent activity against tumor cells. Since *T. annulata* belongs to the same phylum as *Plasmodium* parasites, we have analysed the anti-*Theileria*l activity of ART, ARS, DHART, and ARM against the asexual schizont stage parasites.

Our data shows the potent activity of ARS & DHART at a lower concentration than ART & ARM. Further, we investigated the mechanism of action of ARS and DHART against the parasite-infected cells. We observed a synergistic impact of ARS or DHART on the in vitro activities of BPQ when used in combination. Our results showed rapid killing and reduction of the parasite-infected cells within 12–24 h of treatment with ARS or DHART combined with BPQ or alone. The therapeutic combination of the ARS or DHART with BPQ enhanced the efficacy of the treatment and significantly reduced the effective drug concentrations needed for killing the parasite-infected cells. The ARS and DHART showed minimal toxicity against the mammalian cells. Our data highlights the therapeutic potential of this class of antimalarials against the *Theileria-*infected cells.

Iron-dependent increased ROS levels leading to DNA damage, and protein degradation have been linked to the anti-malarial action of ARS and DHART, respectively [42, 43]. Our results indicate that ARS and DHART treatment leads to cellular damage mediated by increased ROS production in the *Theileria*-infected cells. Increased ROS production cause DNA damage, loss of mitochondrial potential, and caspase-dependent apoptosis in the cells. We found rapid dose-dependent DNA damage as the primary mechanism behind the anti-*Theileria*l activity of the ARS or DHART. According to return to growth experiment results, ARS or DHART therapy causes permanent damage to the infected cells. Overall our data indicate that ARS or DHART induces early and severe DNA damage in parasite-infected cells, resulting in death.

Treatment with ARS has previously been shown to increase anti-tumor activity in cancer cells via ROS-mediated oxidative DNA damage and persistent DSBs in the DNA [39]. However, ferrous iron in some tumor cell lines also has increased the susceptibility to ARS [49]. Since *Theileria* transformed cells show a cancer-like phenotype, we postulate that the anti-parasitic effect of the ARS or DHART is due to ROS-mediated oxidative DNA damage. Next, we have shown that ROS-mediated oxidative DNA damage induced by ARS or DHART treatment is completely reversible by adding ROS scavenger (NALC), confirming its role in the anti-parasitic activity of these drugs. Previous research with *P. falciparum* parasites revealed that important mutations in genes involved in DNA repair pathways have a role in artemisinin resistance [50]. Our results demonstrate the significance of DNA damage and repair pathways during *Theileria* infection and suggest that they may be exploited in developing novel anti-parasitic drugs. Additional studies are warranted to explore the regulatory role of the DNA repair pathway and ROS-mediated oxidative DNA damage during ARS or DHART treatment. ARS is previously shown to induce growth arrest and apoptosis in cancer cells by activating p53-dependent pathways [51, 52]. In *Theileria*-infected cells, ARS or DHART treatment leads to activation and nuclear localization of the p53, suggesting p53-dependent apoptosis.

**Fig. 7:**
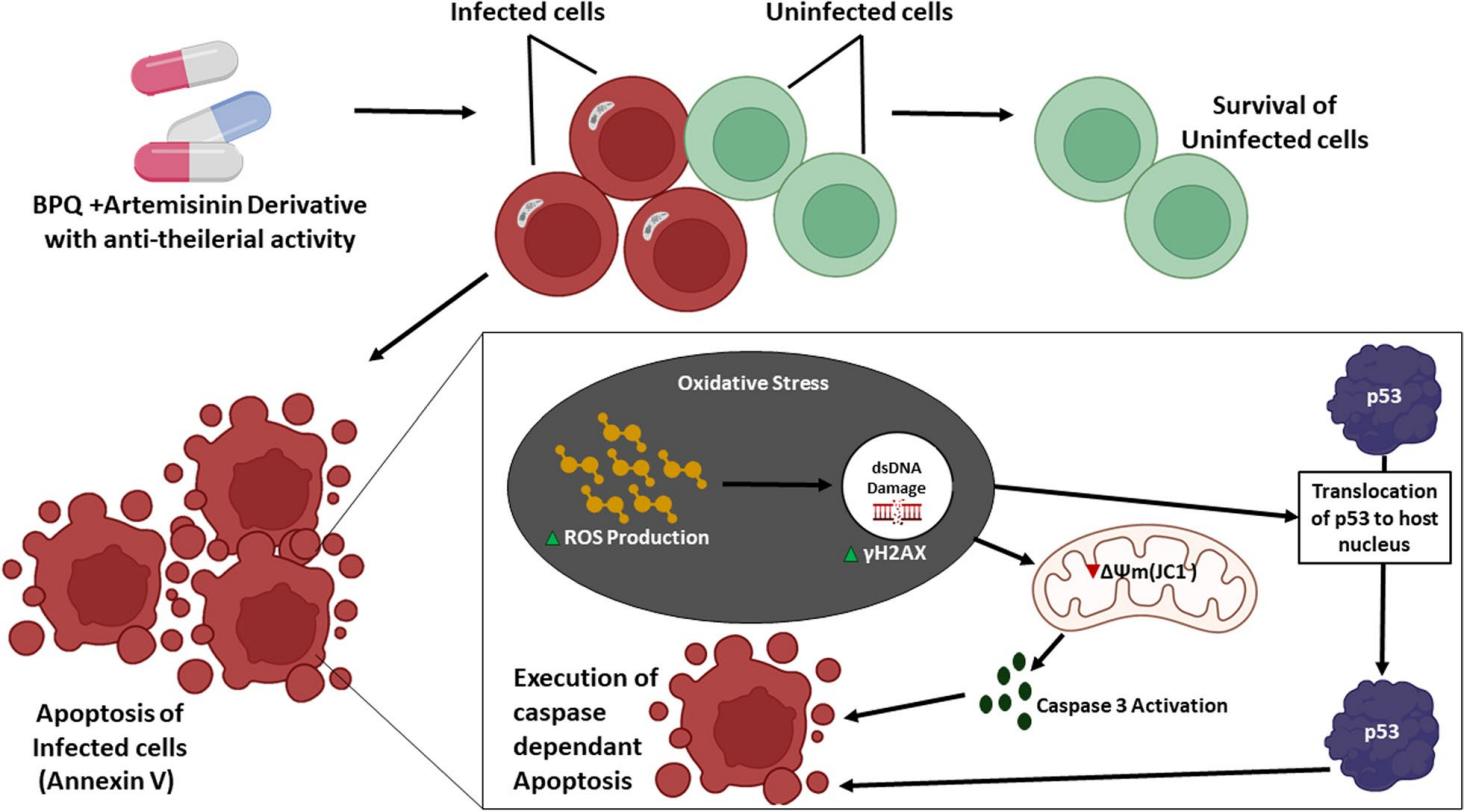
Proposed mechanism of action of ARS and DHART against *T. annulata* parasites: Representative figure shows that treatment with ARS and DHART leads to increased ROS production, which induces oxidative stress and DNA damage causing p53 activation by caspase-dependent apoptosis in the *Theileria* infected cells

In conclusion, our data show that ARS and DHART have potent anti-*Theileria*l activity. The combination therapy enhanced the activity of ARS and DHART synergistically when combined with BPQ. The synergistic action will postpone the emergence of resistance while simultaneously increasing the therapeutic value of the already available drug. We also found that ARS or DHART-induced parasite killing is mediated by ROS-dependent oxidative DNA damage, which initiates p53-dependent apoptotic pathways leading to cell death (Fig. 7). Because ARS and DHART exhibit significant anti-*Theileria*l activity, they offer an alternative to existing therapy and may be employed in the future as a stand-alone therapy or in conjunction with BPQ for the treatment of *Theileria-infected* animals.

## Data Availability

Included in this paper are all datasets created and analysed during this investigation. Additional data may be obtained from the appropriate author upon request.
